# Testicular sperm retrieval at the time of bilateral radical orchiectomy

**Published:** 2010

**Page 22; Column 2; Authors:**

**Anmar M Nassir^1^, John E Grantmyre^2^**

**^1^Department of Surgery, Um Al-Qura University, Makkah, King Faisal Specialist Hospital and Research Center, Jeddah, Saudi Arabia**

**^2^Department of Urology, Dalhousie University, Queen Elizabeth II Health Sciences Center, Halifax NS, Canada**

Should read as

**Anmar M Nassir^1^, John E Grantmyre^2^, Rekha Gupta^3^**

**^1^Department of Surgery, Um Al-Qura University, Makkah, King Faisal Specialist Hospital and Research Center, Jeddah, Saudi Arabia**

**^2^Department of Urology, Dalhousie University, Queen Elizabeth II Health Sciences Center, Halifax NS, Canada**

**^3^Department of Pathology, Dalhousie University, Queen Elizabeth II Health Sciences Center, Halifax NS, Canada**

